# MiRNA-Directed Regulation of VEGF and Other Angiogenic Factors under Hypoxia

**DOI:** 10.1371/journal.pone.0000116

**Published:** 2006-12-27

**Authors:** Zhong Hua, Qing Lv, Wenbin Ye, Chung-Kwun Amy Wong, Guoping Cai, Dayong Gu, Yanhong Ji, Chen Zhao, Jifeng Wang, Burton B. Yang, Yaou Zhang

**Affiliations:** 1 Life Science Division, Graduate School at Shenzhen, Tsinghua University, Shenzhen, China; 2 Sunnybrook Health Sciences Centre, Department of Laboratory Medicine and Pathobiology, University of Toronto, Toronto, Canada; 3 School of Life Science and Technology, Xi'an Jiaotong University, Xi'an, China; 4 Cell and Biochemistry Laboratory, Beijing University of Chinese Medicine, Beijing, China; Centre de Regulació Genòmica, Spain

## Abstract

MicroRNAs (miRNAs) are a class of 20–24 nt non-coding RNAs that regulate gene expression primarily through post-transcriptional repression or mRNA degradation in a sequence-specific manner. The roles of miRNAs are just beginning to be understood, but the study of miRNA function has been limited by poor understanding of the general principles of gene regulation by miRNAs. Here we used CNE cells from a human nasopharyngeal carcinoma cell line as a cellular system to investigate miRNA-directed regulation of VEGF and other angiogenic factors under hypoxia, and to explore the principles of gene regulation by miRNAs. Through computational analysis, 96 miRNAs were predicted as putative regulators of VEGF. But when we analyzed the miRNA expression profile of CNE and four other VEGF-expressing cell lines, we found that only some of these miRNAs could be involved in VEGF regulation, and that VEGF may be regulated by different miRNAs that were differentially chosen from 96 putative regulatory miRNAs of VEGF in different cells. Some of these miRNAs also co-regulate other angiogenic factors (differential regulation and co-regulation principle). We also found that VEGF was regulated by multiple miRNAs using different combinations, including both coordinate and competitive interactions. The coordinate principle states that miRNAs with independent binding sites in a gene can produce coordinate action to increase the repressive effect of miRNAs on this gene. By contrast, the competitive principle states when multiple miRNAs compete with each other for a common binding site, or when a functional miRNA competes with a false positive miRNA for the same binding site, the repressive effects of miRNAs may be decreased. Through the competitive principle, false positive miRNAs, which cannot directly repress gene expression, can sometimes play a role in miRNA-mediated gene regulation. The competitive principle, differential regulation, multi-miRNA binding sites, and false positive miRNAs might be useful strategies in the avoidance of unwanted cross-action among genes targeted by miRNAs with multiple targets.

## Introduction

MicroRNAs (miRNAs) were discovered over a decade ago but only in recent years have they been recognized as one of the major regulatory gene families in cells. As a new family of small non-coding RNA molecules with approximately 22 nucleotides, miRNAs regulate gene expression through translational repression or mRNA degradation in a sequence-specific manner [1]–[4]. They are known to be involved in gene functioning during development, cell proliferation, apoptosis, differentiation, and carcinogenesis [5]–[11]. MiRNA functional identification has become one of the most active research fields in biology.

However, the study on miRNA function has been limited by several obstacles. In addition to the difficulty of accurately predicting their targets and validating these findings, poor understanding of the general principles of gene regulation by miRNAs is a major obstacle. Recently, with the development of new computational algorithms, more and more targets regulated by miRNAs have been predicted [12]–[19]. Along with the accumulation of the knowledge about miRNAs, the complexity of miRNA-mediated gene regulation is gradually emerging. Discovery of the principles of gene regulation by miRNAs would be helpful in the understanding of their highly complex interactions, and in turn, their biological significance.

Some general principles of gene regulation mediated by miRNAs have been predicted by a bioinformatics approach as follows: (1) miRNAs appear to act cooperatively through multiple target sites in one gene by either one or several different miRNAs, and (2) most miRNAs are involved in translational regulation through targeting several genes [15], [20], [21]. However, these principles have yet to be validated. Furthermore, many questions need to be addressed to better understand miRNA-mediated gene regulation, including whether there is competitive action, the opposite of coordinate action, among miRNAs; whether miRNA co-targeted genes can be in functionally related gene groups; whether miRNAs targeting multiple genes could cause unwanted cross-reactions among functionally unrelated genes, and if so, how to avoid these unwanted cross-reactions.

Since angiogenesis is crucial for a wide variety of physiological and pathological processes including development, wound healing, inflammation, and tumor formation, the regulation of angiogenesis is complex and well controlled. Many molecules have been implicated as positive regulators of angiogenesis. Among them, vascular endothelial growth factor (VEGF) is a pivotal angiogenic factor. Its expression is regulated by many factors [22], [23] but it is not clear whether miRNA is involved in VEGF regulation under hypoxia. In this study, we used human CNE cells (from nasopharyngeal carcinoma) as a model to investigate miRNA-directed regulation of VEGF and other angiogenic factors under hypoxia, and to explore the principles of gene regulation by miRNAs.

## Results

### Computational predictions of putative miRNA regulators of VEGF

**Figure 1:**
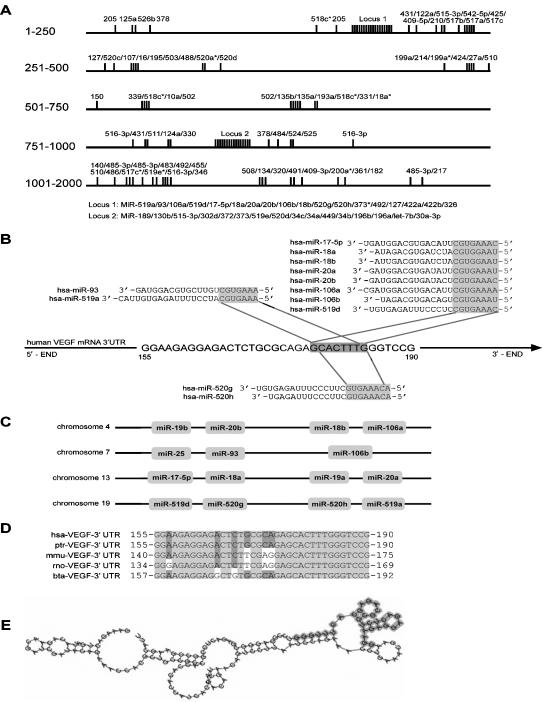
Analysis of miRNA binding sites in VEGF. (A) The map of miRNA binding sites in the VEGF 3′-UTR in accordance with Table S1. Three bioinformatics algorithms, RNAhybrid, miRanda, and FindTar, were used to predict miRNA binding sites in the VEGF 3′-UTR. A binding site map of the VEGF 3′-UTR was generated with the putative binding sites predicted by at least two bioinformatics algorithms. Subsequently, two miRNA binding site-condense regions (MBSCR) were uncovered, that is, 160–195nt and 820–860nt MBSCR. (B) A common miRNA binding site located in 160–185nt shared by 12 miRNAs. (C) The miRNAs that share this common miRNA binding site belong to four different miRNA clusters. (D) The seed region of this common miRNA binding site is highly conserved among mammals. (E) RNAcofold was used to analyze the secondary structure of the common miRNA binding site and its flank regions in the VEGF 3′-UTR. The common binding site is located in an unstable region with a multi-branching loop-like RNA structure.

To investigate whether miRNA is involved in VEGF regulation, we first analyzed miRNA target sites or binding sites in the 3′-untranslated region (3′-UTR) of VEGF and its cognate miRNAs with a bioinformatics approach. Prediction of animal miRNA targets is challenging because there is only a partial pairing of miRNAs with their targets, and the mechanism of miRNA-mediated regulation is poorly understood. To improve the accuracy of binding site prediction, we used three target prediction algorithms, miRanda software [12], RNAhybrid [19], and FindTar, a bioinformatics approach designed in our lab. MiRNAs with binding sites in the VEGF 3′-UTR as predicted by two of the three bioinformatics algorithms were selected as putative regulators of VEGF. In total, 109 binding sites for miRNAs and 96 of their cognate miRNAs were predicted (Table S1). A map of miRNA binding to VEGF 3′-UTR was generated and 2 binding site-condense regions (MBSCR) were observed, including nt160–195 and nt820–860 (Fig. 1A).

In nt160–195 MBSCR, we found a special miRNA binding site at nt160–185, which is shared by 12 miRNAs (Fig. 1B). These miRNAs belong to 4 miRNA clusters (Fig. 1C) and the sequences of the seed region in the binding sites on the VEGF 3′-UTR are highly conserved across all mammals (Fig. 1D). We used RNAcofold (http://www.tbi.univie.ac.at/∼ivo/RNA/) [24] to analyze the secondary structure of the multi-miRNA binding site and its flank regions in the VEGF 3′-UTR and found that the binding sites were located in ‘unstable’ regions with multi-branching loops (Fig. 1E), characteristic of an important miRNA target site. Although it is common for one mRNA to have multi-miRNA binding sites, the results of the computational prediction still surprised us when so many miRNA binding sites and their cognate miRNAs were found targeting VEGF. We then decided to use CNE cells as a cellular system to investigate how miRNAs regulate VEGF expression.

### miRNA and VEGF Expression in CNE cells

Total RNA was obtained for miRNA chip analysis from CNE cells both with and without hypoxia induction. The expression profile of microRNAs in CNE cells is provided as supplementary data (Fig. S1). We compared the expression of miRNAs between the two samples and identified 13 miRNAs that were sharply down-regulated and 6 miRNAs that were greatly up-regulated according to microarray analysis, with fold change≥3 (SAM version 2.1, http://www-stat.stanford.edu/∼tibs/SAM/index.html) [25], [26]. Another 35 miRNAs were also detected in this cellular system, but they did not appear to be differentially expressed between these two samples (Tables 1, 2 and 3). Among these 54 miRNAs, *miR-16, miR-20a, miR-20b, let-7b, miR-17-5p, miR-27a, miR-106a, miR-106b, miR-107, miR-193a, miR-210, miR-320*, and *miR-361* were predicted to target VEGF. *MiR-15b* was also predicted as a putative miRNA regulator of VEGF by one computational algorithm (FindTar).

**Table 1:**
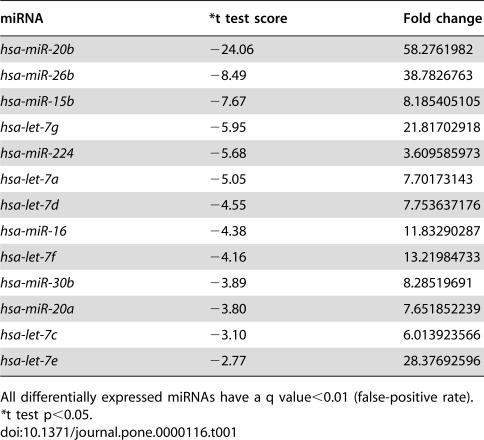
microRNAs down-regulated in DFOM-induced CNE.

miRNA	*t test score	Fold change
*hsa-miR-20b*	−24.06	58.2761982
*hsa-miR-26b*	−8.49	38.7826763
*hsa-miR-15b*	−7.67	8.185405105
*hsa-let-7g*	−5.95	21.81702918
*hsa-miR-224*	−5.68	3.609585973
*hsa-let-7a*	−5.05	7.70173143
*hsa-let-7d*	−4.55	7.753637176
*hsa-miR-16*	−4.38	11.83290287
*hsa-let-7f*	−4.16	13.21984733
*hsa-miR-30b*	−3.89	8.28519691
*hsa-miR-20a*	−3.80	7.651852239
*hsa-let-7c*	−3.10	6.013923566
*hsa-let-7e*	−2.77	28.37692596

All differentially expressed miRNAs have a q value<0.01 (false-positive rate).

* t test p<0.05.

**Table 2:**
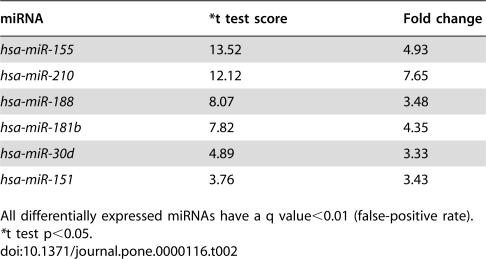
microRNAs up-regulated in DFOM-induced CNE.

miRNA	*t test score	Fold change
*hsa-miR-155*	13.52	4.93
*hsa-miR-210*	12.12	7.65
*hsa-miR-188*	8.07	3.48
*hsa-miR-181b*	7.82	4.35
*hsa-miR-30d*	4.89	3.33
*hsa-miR-151*	3.76	3.43

All differentially expressed miRNAs have a q value<0.01 (false-positive rate).

* t test p<0.05.

**Table 3:**
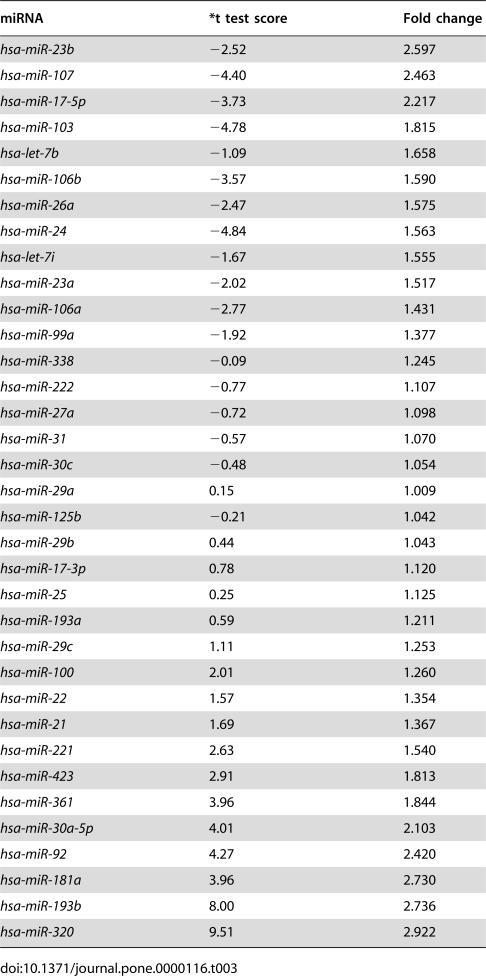
microRNAs non-differentially expressed in CNE with or without hypoxia treatment.

miRNA	*t test score	Fold change
*hsa-miR-23b*	−2.52	2.597
*hsa-miR-107*	−4.40	2.463
*hsa-miR-17-5p*	−3.73	2.217
*hsa-miR-103*	−4.78	1.815
*hsa-let-7b*	−1.09	1.658
*hsa-miR-106b*	−3.57	1.590
*hsa-miR-26a*	−2.47	1.575
*hsa-miR-24*	−4.84	1.563
*hsa-let-7i*	−1.67	1.555
*hsa-miR-23a*	−2.02	1.517
*hsa-miR-106a*	−2.77	1.431
*hsa-miR-99a*	−1.92	1.377
*hsa-miR-338*	−0.09	1.245
*hsa-miR-222*	−0.77	1.107
*hsa-miR-27a*	−0.72	1.098
*hsa-miR-31*	−0.57	1.070
*hsa-miR-30c*	−0.48	1.054
*hsa-miR-29a*	0.15	1.009
*hsa-miR-125b*	−0.21	1.042
*hsa-miR-29b*	0.44	1.043
*hsa-miR-17-3p*	0.78	1.120
*hsa-miR-25*	0.25	1.125
*hsa-miR-193a*	0.59	1.211
*hsa-miR-29c*	1.11	1.253
*hsa-miR-100*	2.01	1.260
*hsa-miR-22*	1.57	1.354
*hsa-miR-21*	1.69	1.367
*hsa-miR-221*	2.63	1.540
*hsa-miR-423*	2.91	1.813
*hsa-miR-361*	3.96	1.844
*hsa-miR-30a-5p*	4.01	2.103
*hsa-miR-92*	4.27	2.420
*hsa-miR-181a*	3.96	2.730
*hsa-miR-193b*	8.00	2.736
*hsa-miR-320*	9.51	2.922

**Figure 2:**
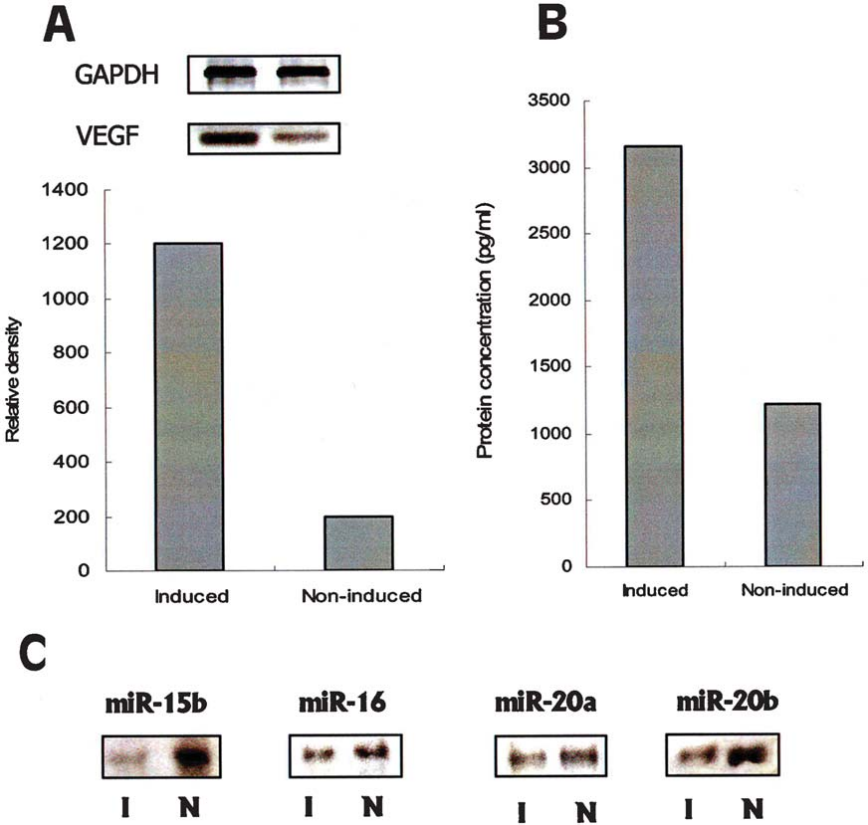
VEGF and miRNA expression in CNE cells. Cell lysate and culture medium from DFOM-induced CNE cells were collected. RT-PCR (A) and ELISA (B) assays were performed to determine VEGF expression at the mRNA and protein levels. CNE cells without DFOM treatment were used as a control. The *mir*Vana™ miRNA Detection procedure was used to validate miRNA chip data. *miR-15b, miR-16, miR-20a*, and *miR-20b* were down-regulated in hypoxia induced CNE cells (C). I: induced, N: non-induced.

As hypoxia is a strong inducer of VEGF, treatment with DFOM, a hypoxia inducer, usually increases VEGF expression [27]. We performed RT-PCR and ELISA assays to determine VEGF expression at the mRNA and protein levels, and found that VEGF was up-regulated in hypoxia-induced CNE cells compared with control cells (Fig. 2A and B). These results were in sharp contrast to the down-regulation of 13 different miRNAs. We hypothesized that some of the miRNAs which were down-regulated in hypoxia-induced CNE cells might unblock VEGF and cause the increase in its expression. In line with this hypothesis, 4 of these 13 down-regulated miRNAs identified by miRNA chip assays were predicted to target VEGF. These are *miR-15b, miR-16, miR-20a*, and *miR-20b*. Down-regulation of these 4 miRNAs in hypoxia-induced CNE cells was confirmed by an miRNA-detecting experiment (Fig. 2C).

### Validation of the repressive effect of miRNA regulators on VEGF expression

**Figure 3:**
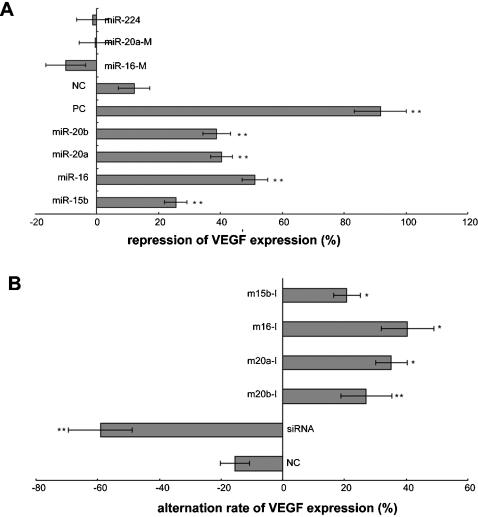
Validation of the repressive effect of putative miRNA regulators on VEGF expression in CNE cells. (A) Transient overexpression of miRNAs in hypoxia-induced CNE cells. The effect of *miR-15b, miR-16, miR-20a*, and *miR-20b* on VEGF expression was tested in CNE cells by transfection of the cells with siRNA duplexes homologous in sequence to the miRNAs. ELISA was used to detect VEGF expression levels. The controls consist of one positive control: VEGF-siRNA (PC), and four negative controls: *miR-224*, mutated *miR-16*, and *miR-20a* (*miR-16M, miR-20aM*), and a random sequence (NC). (B) Transfection of miRNA inhibitors in hypoxia non-induced CNE cells. CNE cells were transfected with inhibitors of* miR-15b, miR-16, miR-20a*, and *miR-20b*. siRNA against VEGF was used as positive control. ELISA was used to detect the change in VEGF expression levels after endogenous *miR-15b, miR-16, miR-20a*, and *miR-20b* were inhibited. *, p<0.05; **, p<0.01. NC: negative control; PC: positive control.

To investigate the putative effects of *miR-15b, miR-16, miR-20a*, and *miR-20b*, we determined the consequence of over-expressing these miRNAs on VEGF expression. Hypoxia-induced CNE cells, which expressed high levels of VEGF but lacked *miR-15b, miR-16, miR-20a*, and *miR-20b*, were transfected with synthetic miRNA duplexes of these miRNAs and a set of controls. These include one positive control, VEGF-siRNA, and four negative controls, *miR-224*, mutated *miR-16* (*miR-16M* 5′-UAGCCUAACGUAAAUAUUGGCG- 3′) and *miR-20a, miR-20aM* 5′ -UACGUUGCUUAUAGUGCAGGUAG- 3′), and a random sequence. *MiR-224* was chosen as one of the negative controls because it was down-regulated during hypoxia but was not predicted to target VEGF. Transfection with *miR-15b, miR-16, miR-20a*, and *miR-20b*, but not the negative controls, resulted in a 26–51% decrease in VEGF expression at the protein level 30h after transfection (Fig. 3A).

We also analyzed the effect of inhibiting endogenous *miR-15b, miR-16, miR-20a*, and *miR-20b* on VEGF expression. Using the same transfection method, we introduced inhibitors of these miRNAs into normoxic CNE cells, which express low levels of VEGF and high levels of endogenous *miR-15b, miR-16, miR-20a*, and *miR-20b*. As we expected, VEGF expression measured by ELISA increased significantly (Fig. 3B).

**Figure 4:**
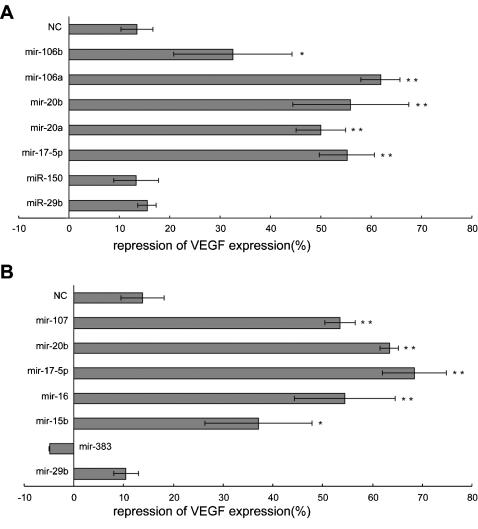
Luciferase activity assay. Luciferase reporter constructs were generated through placing fragments of 185 bp (Construct I, sequence from nt31–216 of the VEGF 3′-UTR) and 241 bp (Construct II, sequence from nt703–944 of the VEGF 3′-UTR) at the 3′ end of luciferase gene in pRL-TK. COS-7 cells were co-transfected with a luciferase reporter vector and an miRNA which has a putative binding site in either Construct I (A) or Construct II (B). Luciferase activity was measured to determine the effects of these miRNAs on luciferase translation. In addition to a random sequence (NC), *miR-29, miR-150*, and *miR-383* which have putative binding sites in the 3′-UTR of VEGF but not on Construct I or II, as predicted by all of the algorithms, were employed as negative controls. *, p<0.05; **, p<0.01.

To demonstrate a direct interaction between the 3′-UTR of VEGF with candidate miRNAs, we inserted two fragments of the VEGF 3′-UTR predicted to interact with these miRNAs into a luciferase expression vector to generate luciferase reporter constructs. Construct I contained a fragment located in nt31–216 of the VEGF 3′-UTR, while Construct II contained a fragment in nt703–944 of the VEGF 3′-UTR. Computational predictions indicated that *miR-20a, miR-20b, miR-17-5p, miR-106a*, and *miR-106b* had binding sites in Construct I. With slightly relaxed criteria about free energy and conservation, *miR-15b, miR-16, miR-17-5p, miR-20b*, and *miR-107* have computationally predicted target sites in Construct II reporters (Table 4). In addition to a random sequence, *miR-29b, miR-150*, and *miR-383* were employed as negative controls. All three of these miRNAs have putative binding sites predicted by one at least of the three algorithms on the 3′UTR of VEGF but *miR-29b* and *miR-150* do not have binding sites on Construct I, and *miR-29b* and *miR-383* have no binding sites on Construct II. These miRNAs were co-transfected with their respective reporters into COS-7 cells and the levels of luciferase activity were measured to determine the repressive effects of these miRNAs. The dual-luciferase activity assays revealed a repression of 32–68% of luciferase activity compared to the controls, which showed a repression of less than 15% (Fig. 4A and B).

**Table 4:**
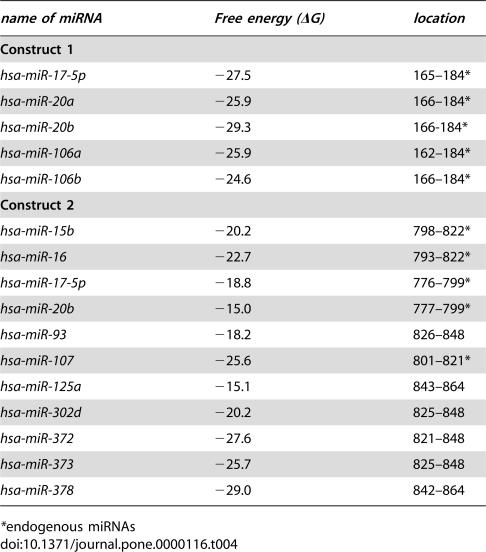
miRNA binding sites at fragments of the VEGF 3′-UTR in report vector Construct I or II.

*name of miRNA*	*Free energy (ΔG)*	*location*
**Construct 1**		
*hsa-miR-17-5p*	−27.5	165–184*
*hsa-miR-20a*	−25.9	166–184*
*hsa-miR-20b*	−29.3	166-184*
*hsa-miR-106a*	−25.9	162–184*
*hsa-miR-106b*	−24.6	166–184*
**Construct 2**		
*hsa-miR-15b*	−20.2	798–822*
*hsa-miR-16*	−22.7	793–822*
*hsa-miR-17-5p*	−18.8	776–799*
*hsa-miR-20b*	−15.0	777–799*
*hsa-miR-93*	−18.2	826–848
*hsa-miR-107*	−25.6	801–821*
*hsa-miR-125a*	−15.1	843–864
*hsa-miR-302d*	−20.2	825–848
*hsa-miR-372*	−27.6	821–848
*hsa-miR-373*	−25.7	825–848
*hsa-miR-378*	−29.0	842–864

* endogenous miRNAs

### Combinatorial regulation by miRNAs

**Figure 5:**
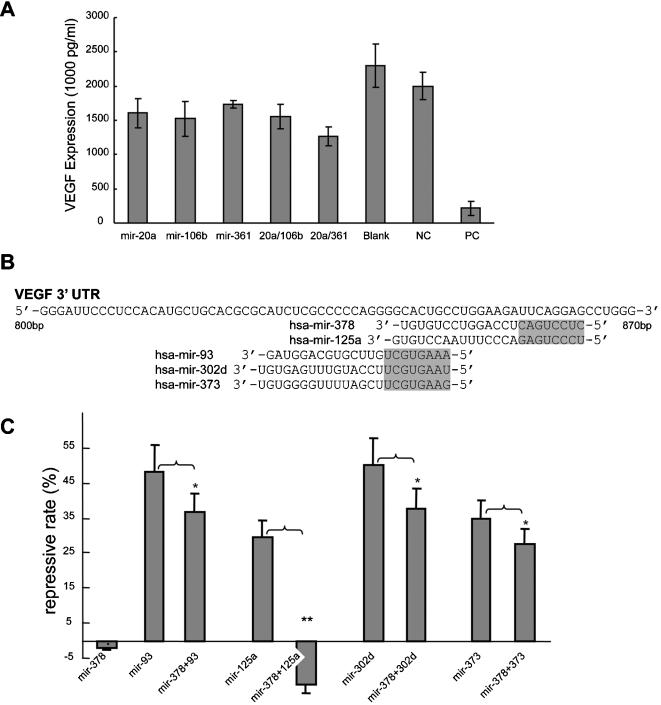
Effects of different combinations of miRNAs on VEGF expression. (A) CNE cells were transfected or co-transfected with *miR-20a* and *miR-106b*, which share the same binding site, or with *miR-20a* and *miR-361*, which target different binding sites. An unrelated random sequence was used as a negative control (NC). A specific sequence targeting VEGF (functioning as siRNA) was used as a positive control (PC). Culture medium was collected 30h after transfection. Repression of VEGF expression was detected by ELISA. (B) miRNA binding site locations of *miR-378, miR-125a, miR-93, miR-302d*, and *miR-373* in the VEGF 3′-UTR fragment of Construct II. (C) COS-7 cells were co-transfected with (1) Construct II and *miR-378, miR-125a, miR-93, miR-302d*, or *miR-373*; (2) Construct II and *miR-378* plus either *miR-125a, miR-93, miR-302d*, or *miR-373*. Luciferase activity was measured to investigate the effects of different combinations of miRNAs. *, p<0.05; **, p<0.01.

We have shown that VEGF expression is regulated by multiple miRNAs. Some miRNAs shared a common binding site, whereas other miRNAs have their own binding sites in the 3′-UTR of VEGF. To investigate whether different combinations of miRNAs have different contributions towards VEGF regulation, we performed co-transfection experiments using *miR-20a* with *miR-106b*, which share the same binding site, and *miR-20a* with *miR-361*, which target their own binding sites. Compared to single transfection with *miR-20a, miR-106b*, or *miR-361*, co-transfection of *miR-20a* with *miR-361* exhibited additive repression on VEGF expression, suggesting a coordinate action of these miRNAs (Fig. 5A). On the other hand, little difference was observed between co-transfection of cells with *miR-20a* and *miR-106b*. These results suggest that different miRNA combination patterns can produce coordinate action, which increases the repressive effect of miRNAs on VEGF translation, or competitive action, which failed to generate further repression of VEGF translation. The result of coordinate action is consistent with Krek's report [20].

Competitive action among miRNAs can weaken the total repressive power of an miRNA group. We observed this effect when miRNAs already validated as true regulators of VEGF were co-transfected with an miRNA validated as a false positive regulator. In luciferase reporter Construct II, the inserted fragment of VEGF 3′-UTR contains 1 putative targeting site for *miR-378,* located at nt842–864. Four other miRNAs (*miR-93, miR-125a, miR-302d*, and *miR-373*) also targeted this region. The binding sites of these 5 miRNAs overlapped each other (Fig. 5B). In luciferase activity assays, a significant repressive effect was detected when COS-7 cells were co-transfected with Construct II and *miR-93, miR-125a, miR-302d*, or *miR-373*, but not *miR-378* (Fig. 5C). However, when cells were co-transfected with *miR-378* and one of these four miRNAs, the repressive effect of these miRNAs decreased significantly. In the case of *miR-125a*, which shares the same seed region with *miR-378*, the repressive activity of *miR-125a* was abolished completely, suggesting a competitive interaction between these two miRNAs. Our results also suggest that some of the false positive miRNAs of a gene may be involved in miRNA-mediated gene regulation through competitive action. This may be useful to prevent over-repression of functional miRNAs.

### Co-regulatory effect of *miR-15b, miR-16, miR-20a*, and *miR-20b* on angiogenic factors in CNE cells

When under hypoxia stimulation, VEGF is not the only angiogenic gene up-regulated, as other angiogenic factors have been reported to be up-regulated as well [22], [23]. Usually, these factors cooperate together resulting in angiogenesis. We hypothesize that there must be common regulatory mechanism(s) to modulate multiple factors involved in angiogenesis. MiRNAs with multiple targets are able to serve the purpose of co-regulating a group of functionally related genes, such as multiple angiogenic factors under strict spacio-temporal conditions.

**Figure 6:**
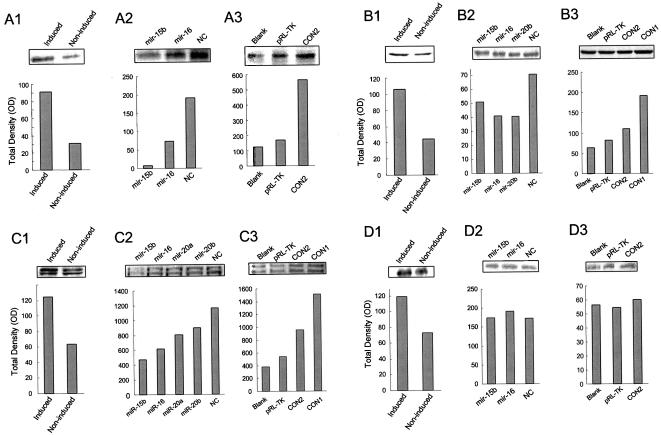
Co-regulation of miRNAs to angiogenic factors. CNE cells were induced with or without DFOM (6–1), transfected with *miR-15b, miR-16, miR-20a*, or *miR-20b* (6–2) and transfected with Construct I or Construct II (6–3). Cell lysate was collected, and the expression of angiogenic factors uPAR (A), COX2 (B), c-MET (C), and PTN (D) were determined by Western Blotting.

To test our hypothesis, we identified the angiogenic factors which are differentially expressed in hypoxia induced CNE cells. In our previous assays of gene expression profiling in CNE cells, 7 of 40 angiogenesis-related genes, *VEGF, c-MET, COX2, uPAR, PAI1, MAPK7*, and *Ang* were up-regulated when CNE cells were treated with DFOM. With computational analysis, *miR-15b* and *miR-16* were predicted to be putative regulatory miRNAs of *uPAR, COX2*, and *c-MET*, in addition to *VEGF. MiR-20a* and *miR-20b* may target *c-MET*, whereas *miR-20b* also targets *COX2*. We further investigated whether these angiogenic factors are co-regulated by these miRNAs, which have been validated as regulators of VEGF in CNE cells. CNE cells were transfected with these miRNAs and with reporter vectors, Construct I, Construct II, and pRL-TK. Western blotting was used to measure protein expression levels of *uPAR, COX2*, and *c-MET. MiR-15b* and *miR-16* down-regulated *uPAR*, whereas Construct II up-regulated its expression. It is probable that the fragment of VEGF 3′-UTR in Construct II competed with *uPAR* for endogenous *miR-15b* and *miR-16* (Fig. 6A). The expression of *COX2* was down-regulated by *miR-15b, miR-16*, and *miR-20b*, and *c-MET* was down-regulated by all three of these miRNAs plus *miR-20a*. Both of these two genes were up-regulated by Construct I and Construct II (Fig 6B and C). To examine the specificity of these miRNAs in the regulation of angiogenic factors expressed in CNE cells, we chose *PTN* (Pleiotrophin) as a negative control. *PTN* is an angiogenic factor known to change during hypoxia, but is not predicted to be a target of either *miR-15b* or *miR-16*. First we confirmed that *PTN* expression was up-regulated by the stimulation of hypoxia in CNE cells (Fig. 6D). Then we transfected CNE cells with *miR-15b* and *miR-16*, and found that *miR-15b* and *miR-16* did not have a repressive effect on *PTN* expression. Furthermore, we used Construct II to compete with *PTN* for endogenous *miR-15b* and *miR-16*, but Construct II could not up-regulate *PTN* expression. This was expected, as no binding site for *miR-15b* or *miR-16* was detected by either the miRanda software, RNAhybrid, or FindTar algorithms in the 3′-UTR fragment of *PTN*. These data indicate that some of angiogenic factors expressed in CNE cells were specifically co-regulated by *miR-15b, miR-16, miR-20a*, and *miR-20b*.

## Discussion

### Possible mechanisms for changes in miRNA levels during hypoxia in CNE cells

In this investigation, we found that *miR-15b, miR-16, miR-20a*, and *miR-20b* are sharply down-regulated in CNE cells after hypoxia treatment. The mechanisms for these changes are not clear; however, we might find some clues from changes in transcription factors during hypoxia. The tumor suppressor gene p53 might be one of the critical factors which cause the changes in miRNA levels during hypoxia. Previous studies indicate that hypoxia could induce the accumulation of p53 [28], [29], and it is possible that the accumulation of p53 may regulate the expression of several miRNAs. As an example, *miR-15b* was down-regulated significantly in hypoxic conditions. While no p53-binding sites have been predicted in the *miR-15b* promoter [30], it is possible that p53 down-regulates *miR-15b* expression through an indirect way. Since both *miR-15b* and one of two *miR-16* genomic loci, *miR-16-2*, are located in the same cluster on chromosome 3 (the *miR-15b* cluster), the expression of *miR-16* may be regulated by the same mechanism as that of *miR-15b*. In contrast to *miR-15b* and *miR-16*, the down-regulation of *miR-20a* and *miR-20b* may be related to hypoxia inducible factor-1α (HIF-1α). In response to hypoxia, HIF-1α becomes stabilized and able to counteract c-Myc functionally. In keeping with its antagonism of Myc, HIF-1α down-regulates c-Myc-activated genes such as hTERT, BRCA1 [31], [32], and might also down-regulate *miR-20a* and *miR-20b*, since both *miR-20a* and *miR-20b* may be c-Myc-activated genes. As a member of the *miR-17* cluster located on chromosome 13, the expression of *miR-20a* is regulated by c-Myc through direct binding to the *miR-17* cluster locus. Up-regulation of *miR-20b*, a member of the *miR-106a* cluster located on X chromosome, was also consistently observed in the high c-Myc state and seven putative binding sites in the vicinity of the *miR-106a* cluster were also identified even though no direct binding between c-Myc and these binding sites has been reported [33]. Taken together, the down-regulation of *miR-15b, miR-16, miR-20a*, and *miR-20b* in CNE cells might be mediated by the accumulation of p53 or the stabilization of HIF-1α during hypoxia.

### Principles of gene regulation by miRNA

Initial data released by this investigation first reported miRNA-directed VEGF regulation. Through analysis of this data, we uncovered some new principles of gene regulation by miRNA and also further validated principles predicted by others [15], [20], [21] using experimental methods. The biological significance of these principles was also addressed in this investigation.

The first is the coordinate principle. John and Marks [15] proposed that miRNAs may act cooperatively through multiple target sites in one gene. Krek's report proved this principle [20] and our results further indicate that miRNAs with independent binding sites in a gene can produce coordinate action, which increases the repressive effect of miRNAs on translation of this gene.

The second is the co-regulatory principle. It is a common phenomenon that groups of functionally related genes act together to regulate a physiologically related function (e.g. angiogenesis). It therefore appears as if a common regulatory mechanism controls the multiple factors involved in these functions. MiRNA with the ability to target multiple genes could potentially regulate a group of functionally related genes. In this study, we determined that some miRNAs identified as VEGF regulators also regulated the expression of other angiogenic factors. We call this regulatory pattern a co-regulatory principle, meaning that one or a few miRNAs can co-regulate a group of functionally related genes. This is a convenient and efficient regulatory pattern but may cause unwanted cross-reaction between genes, if an miRNA is expressed for regulating one gene, but also targets other genes which need not be repressed under the same conditions. Therefore, there must be some mechanisms to prevent unwanted cross-reactions.

The third is the principle of differential regulation. As an miRNA-targeted gene, VEGF might be regulated by many miRNAs, according to the number of binding sites in its 3′-UTR. However, only some of these miRNAs are involved in VEGF regulation or VEGF maintenance in CNE cells. The others might be reserved for the needs of other cells under different conditions. To ensure that VEGF expression is regulated by distinct miRNAs in different cells under different conditions, we analyzed the relationship between VEGF expression and the miRNA expression profile in five VEGF-expressing cell lines: CNE, HeLa [34], MCF-7, HL60, and K562 [35]. In these cell lines, 31–104 miRNAs were detected and 9–31 of those miRNAs were predicted as putative regulators of VEGF. Through comparison of these five cell lines, we found that not only does each cell line express different numbers of miRNAs, they also employ discrete combinations of VEGF-related miRNAs (Table 5). Since angiogenesis is crucial for a wide variety of physiological and pathological processes including development, wound healing, inflammation, and tumor formation, many molecules have been implicated as positive regulators of angiogenesis. Being a pivotal factor, VEGF expression is complex and needs to be well regulated [22], [23]. Various miRNA binding sites provide a number of choices which not only meet different needs under various processes but also avoid unwanted cross-reactions. We call this pattern differential regulation, which means that a gene with multiple binding sites for a number of miRNAs can be regulated by discrete miRNAs in differing cells under different conditions. However, the pattern of differential regulation may not be enough to prevent unwanted cross-reactions between genes, since some miRNAs have too many target genes.

**Table 5:**
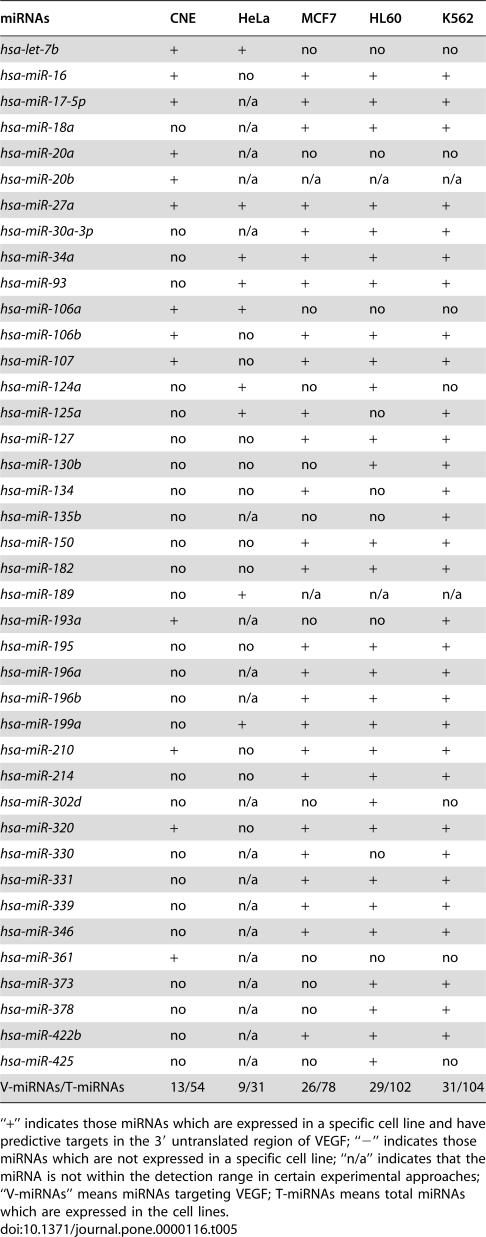
miRNAs which putatively target VEGF and are expressed in CNE, HeLa, MCF7, HL60, and K562.

miRNAs	CNE	HeLa	MCF7	HL60	K562
*hsa-let-7b*	+	+	no	no	no
*hsa-miR-16*	+	no	+	+	+
*hsa-miR-17-5p*	+	n/a	+	+	+
*hsa-miR-18a*	no	n/a	+	+	+
*hsa-miR-20a*	+	n/a	no	no	no
*hsa-miR-20b*	+	n/a	n/a	n/a	n/a
*hsa-miR-27a*	+	+	+	+	+
*hsa-miR-30a-3p*	no	n/a	+	+	+
*hsa-miR-34a*	no	+	+	+	+
*hsa-miR-93*	no	+	+	+	+
*hsa-miR-106a*	+	+	no	no	no
*hsa-miR-106b*	+	no	+	+	+
*hsa-miR-107*	+	no	+	+	+
*hsa-miR-124a*	no	+	no	+	no
*hsa-miR-125a*	no	+	+	no	+
*hsa-miR-127*	no	no	+	+	+
*hsa-miR-130b*	no	no	no	+	+
*hsa-miR-134*	no	no	+	no	+
*hsa-miR-135b*	no	n/a	no	no	+
*hsa-miR-150*	no	no	+	+	+
*hsa-miR-182*	no	no	+	+	+
*hsa-miR-189*	no	+	n/a	n/a	n/a
*hsa-miR-193a*	+	n/a	no	no	+
*hsa-miR-195*	no	no	+	+	+
*hsa-miR-196a*	no	n/a	+	+	+
*hsa-miR-196b*	no	n/a	+	+	+
*hsa-miR-199a*	no	+	+	+	+
*hsa-miR-210*	+	no	+	+	+
*hsa-miR-214*	no	no	+	+	+
*hsa-miR-302d*	no	n/a	no	+	no
*hsa-miR-320*	+	no	+	+	+
*hsa-miR-330*	no	n/a	+	no	+
*hsa-miR-331*	no	n/a	+	+	+
*hsa-miR-339*	no	n/a	+	+	+
*hsa-miR-346*	no	n/a	+	+	+
*hsa-miR-361*	+	n/a	no	no	no
*hsa-miR-373*	no	n/a	no	+	+
*hsa-miR-378*	no	n/a	no	+	+
*hsa-miR-422b*	no	n/a	+	+	+
*hsa-miR-425*	no	n/a	no	+	no
V-miRNAs/T-miRNAs	13/54	9/31	26/78	29/102	31/104

“+” indicates those miRNAs which are expressed in a specific cell line and have predictive targets in the 3′ untranslated region of VEGF; “−” indicates those miRNAs which are not expressed in a specific cell line; “n/a” indicates that the miRNA is not within the detection range in certain experimental approaches; “V-miRNAs” means miRNAs targeting VEGF; T-miRNAs means total miRNAs which are expressed in the cell lines.

Competitive action was recognized as the final principle of gene regulation by miRNAs, and it can be seen when multiple miRNAs compete with each other for a common binding site (called a multi-miRNA binding site) or a functional miRNA competes with a false positive miRNA for the same binding site. The biological role of competitive action is not very clear. It might be related to prevention of unwanted cross-reactions between genes. In this investigation, we found a multi-miRNA binding site located in nt162–185 of the VEGF 3′-UTR, which functioned as a specially designed ruse for the prevention of unwanted cross-reactions between miRNA-targeting genes. This binding site is shared by 12 different miRNAs, according to the bioinformatics prediction, but only *miR-17-5p, miR-20a, miR-20b, miR-106a*, and *miR-106b* were detected in DFOM-untreated CNE cells. Among these 5 miRNAs, *miR-20a* and *miR-20b* were validated as regulatory miRNAs in hypoxia-induced VEGF expression, whereas the other three could be expressed for the regulation of other genes without over-repressing VEGF expression due to competitive action. Thus, the unwanted cross-reaction of VEGF with other genes could be prevented. In some circumstances, if VEGF expression needs no repression, but some regulatory miRNAs of VEGF are expressed for regulation of other genes, the unwanted cross-reaction of VEGF can be decreased through the expression of false positive miRNAs to compete with the functionally effective miRNAs. The competitive principle, differential regulation, multi-miRNA binding sites, and false positive miRNAs might be useful strategies to avoid unwanted cross actions between targeting genes of miRNAs with multiple targets. The patterns of miRNA-directed VEGF regulation demonstrate several principles of miRNA-mediated gene regulation, and these principles will be helpful for us to understand the highly complex pattern of gene regulation by miRNAs.

### Some concerns about negative control miRNAs

To test the specificity of prediction of miRNA target sites, we first did experiments with a luciferase activity assay to test: a) the effects of *miR-106a* and *miR-106b* on Construct II; and b) the effects of *miR-15b* and *miR-16* on Construct I. We found that all of these miRNAs showed repression of 20–27% of luciferase activity. The criteria that we used to predict miRNA targets of VEGF in this investigation are: only the binding sites predicted by two of the three bioinformatics algorithms, miRanda software, RNAhybrid, and FindTar algorithm, were selected as putative binding sites for miRNA in the VEGF 3′-UTR. According to the criteria, no binding sites for *miR-15b* and *miR-16* on Construct I and *miR-106a* and *miR-106b* on Construct II were found. However, the result of the luciferase activity assay suggests that this is a false negtive prediction. Combinative use of more than one computational algorithm in the prediction of miRNA binding sites could decrease the false positive rate, but it might increase the false negtive rate because the overlap between computational algorithms is not very good. When the prediction was carried out with miRanda software, RNAhybrid, and FindTar algorithms separately, binding sites for *miR-15b* and *miR-16* on Construct I and *miR-106a* and *miR-106b* on Construct II would be detected by one of these algorithms. We therefore chose *miR-29b, miR-150*, and *miR-383*, which have putative binding sites on the 3′-UTR of VEGF but do not have putative binding sites on Construct I or II, as predicted by all of the three algorithms, to test the specificity. As we expected, *miR-29b, miR-150*, and *miR-383* did not show repressive effects on Construct I or II. Our results suggest that it is important to confirm that an miRNA is not a potential regulatory miRNA of a specific gene by using different bioinformatics algorithms before using the miRNA as a negative control.

## Materials and Methods

### Cell culture and hypoxia inducement

CNE cells, a human nasopharyngeal carcinoma cell line, were obtained from Kunming Cell Bank (Kunming, China) and maintained in RPMI 1640 medium containing 10% FBS at 37°C with 5% CO_2_. Hypoxia inducement was carried out with DFOM (Sigma-Aldrich Co., MO, USA) at a final concentration of 130 uM and cells without DFOM treatment were used as control. After 20 hours of DFOM treatment, the cells were harvested and total RNA was isolated with TRIzol Reagent (Invitrogen Corp. CA) according to the manufacturer's protocol.

### RNA preparation, miRNA microarray experiments, and miRNA detection

Total RNA samples were sent to CapitalBio (CapitalBio Corp., Beijing, China) for miRNA microarray experiments. Procedures were performed as described in detail on the website of CapitalBio (http://www.capitalbio.com). Briefly, 50–100 µg of total RNA were used to extract miRNA using an miRNA Isolation Kit (Ambion Inc. Texas). Fluorescein-labeled miRNA [36] were used for hybridization on each miRNA microarray chip containing 509 probes in triplicate, corresponding to 435 human (including 122 predicted miRNAs), 261 mouse, and 196 rat miRNAs found in the miRNA Registry (http://microrna.sanger.ac.uk/sequences/; accessed Oct. 2005) or collected from a published paper [37]. Raw data were normalized and analyzed in GenePix Pro 4.0 software (Axon Instruments, PA, USA). Expression data were median-centered by using the global median normalization of the BIOCONDUCTOR package (http://www.bioconductor.org). Statistical comparisons were done with SAM software (SAM version 2.1, http://www-stat.stanford.edu/∼tibs/SAM/index.html) [25], [26]. The microarray data were confirmed through an miRNA detection experiment with the *mir*Vana™ miRNA detection kit (Ambion Inc. Texas).

### Cell transfection with miRNAs or miRNA inhibitors

SiRNA duplexes homologous in sequence to miRNA and their inhibitors were synthesized and purified by Shanghai GenePharma Co. (Shanghai, China). The sequences of these inhibitors are the exact antisense copy of the mature miRNAs and all the nucleotides in the inhibitors contain 2′-OMe modifications at each base. A VEGF siRNA which targets mRNA sequence 5′-GGAGUACCCUGAUGAGAUC-3′ (nt189–207) [38] was used as a positive control (PC). SiRNA duplexes with non-specific sequences were used as a negative control (NC).

MiRNA duplexes or their inhibitors were transfected into CNE cells using the transfection protocol recommended by Invitrogen. In brief, CNE cells were seeded into 96 or 24 well plates one day prior to transfection. When the cells reached 50–70% confluence, miRNA duplexes or their inhibitors were transfected into the cells at a concentration of 4 or 6 pmol/well for the 96 well plates, and 20 or 30 pmol/well for the 24 well plates, using the Lipofectamine 2000 reagent (Invitrogen Corp. CA, USA). Hypoxia induction was carried out using the method described before. The same amount of negative control RNA duplexes and VEGF siRNA were also transfected. The cell lysate and culture medium were collected 30 hr post-transfection.

### Assay of luciferase activity

To perform luciferase activity assays, VEGF 3′-UTR (nt163–184) and its flank sequence was PCR-amplified using two primers, 5′-CGTTCTAGAGTTTCGGGA ACCAGATCTC-3′ and 5′-AACACTAGTAATGCTTCCGCCGGAGT-3′. Two copies of the target sequence were cloned downstream of the stop codon in pRL-TK (Promega Corp. WI, USA). The other sequence which contains multiple binding sites in VEGF 3′-UTR (nt700–850) was also amplified using two primers, 5′- TCTTCTAGACAGGTCAGACGGACAG-3′ and 5′-ACAACTAGTCTCTTCTCTTCGCCGG-3′, and two copies of the target sequence were cloned downstream of the stop codon in pRL-TK. We pre-plated COS-7 or CNE cells 24 h prior to transfection in 24-well tissue culture plates. The next day COS-7 or CNE cells were transfected with 100 or 400 ng of pRL-TK (Rr-luc) and 5 or 100 ng of pGL3 control vector (Pp-luc, from Promega Corp. WI, USA). For the co-transfection of miRNAs or their inhibitors with reporter vectors, 20 nM of each miRNA duplex or 30 nM of each inhibitor was transfected using Lipofectamine 2000. Cell lysate was collected and assayed 30 h after transfection. Firefly and *Renilla* luciferase activities were measured using a Dual Luciferase Reporter Assay System (Promega Corp. WI, USA) and each transfected well was assayed in triplicate as described [14].

### ELISA assays

The culture medium of CNE cells was recovered for ELISA assays using VEGF ELISA from R & D systems (Minneapolis, MN) and a GENios ELISA plate reader (TECAN, Austria). All experiments were repeated four times.

### Western blots

Cell supernatant or cell lysate was subjected to SDS-PAGE and transferred to a nitrocellulose membrane. Protein expression was analyzed by Western blotting with primary antibodies against uPAR, COX2, c-MET, and PTN (Santa Cruz, California) and then incubated with a secondary antibody. After washing, the bound antibody was visualized with an ECL kit (Amersham Pharmacia Biotech, NJ, USA) as described previously [39].

### RT-PCR

Total RNA was isolated with TRIzol Reagent (Invitrogen, Gaithersburg, MD) according to the manufacturer's protocol. RT-PCR was performed with the total RNA using TaKaRa one step RNA PCR kit (TaKaRa Bio Inc, Japan). The VEGF expression level was detected with a pair of primers, forward: 5′-GAGGGCAGAATCATCACGAA-3′; reverse: 5′-GGGAACGCTCCAGGACTTAT-3′. The gene expression level of VEGF was normalized against a housekeeping gene, glyceraldehyde-3-phosphate dehydrogenase (GADPH).

### Bioinformatics

miRNA target site prediction of VEGF and other angiogenic factors was performed by using miRanda software [12] (http://www.microrna.org/miranda_new.html), RNAhybrid [19] (http://bibiserv.techfak.uni-bielefeld.de/rnahybrid/webservice.html), and FindTar algorithm, a bioinformatics approach designed by us. MiRanda was used for the primary screening of miRNA target sites with cut-off values for free energy (ΔG)<−14 kcal/mole and scores>70, and the FindTar algorithm for secondary screening with a criteria of (ΔG)<−20 kcal/mole, and high conservation among mammals. The program was run with a temperature setting at 37°C.

#### 3′-UTR Datasets and MicroRNA Datasets

3′ untranslational region (3′-UTR) sequences of VEGF from human (*Homo sapiens*), mouse (*Mus musculus*), rat (*Rattus norvegicus*), chimpanzee (*Pan troglodytes*), and Cow (*Bos taurus*) were retrieved using Ensembl Data base (http://www.ensembl.org). Human miRNA sequences of the RFAM miRNA registry [40] were downloaded from the miRBase website (http://microrna.sanger.ac.uk/sequences/index.shtml).

#### The design of FindTar algorithm

It was recognized that the nucleus or seed, typically a perfectly Watson-Crick–base-paired stretch of 7 nt starting at either the first or the second base of the microRNA (counted from the 5′ end), was the most critical for target recognition [13], [14], [17]. In the design of the FindTar algorithm, we started with experimental results from other groups [14], [18], and used a slightly relaxed seed criterion to search the mRNA sequences for a binding site, so that more potential miRNA targets could be included in the primary screening. Our seed criterion was defined as a perfectly Watson-Crick–base-paired stretch of 6 nt starting at the first, second, or third base of the miRNA, but one G-U wobble in the seed would be tolerable. After the seed regions were identified, we predicted the secondary structure and minimum free energy of the possible miRNA:miRBS (miRBS: miRNA binding site) using RNAcofold software (Vienna RNA Package 1.5 beta version, http://www.tbi.univie.ac.at/∼ivo/RNA/). RNAcofold was also incorporated into the FindTar algorithm. Sequences of miRNA binding sites in mRNA, and a given miRNA, were input for precisely predicting the RNA secondary structures of the miRNA/miRBS duplexes and calculating minimum free energy (ΔG) of the whole miRNA/miRBS duplex.

## Supporting Information

Table S1 RNAhybrid, miRanda, and FindTar were used to predict potential binding sites of each human miRNA. The sites found by two or all of these three software were chosen as putative binding sites for miRNAs (all these sites were predicted by FindTar). Free energy is calculated by RNAcofold.
(0.14 MB DOC)

Figure S1 Image of miRNA microarray. miRNA array analysis was performed with mRNA samples from hypoxia-induced CNE cells (A and B) and non-induced CNE cells (C and D). An miRNA microarray chip containing 509 probes in triplicate was used in the analysis.
(6.39 MB TIF)
