# Supplementary material for: MiRNA-Directed Regulation of VEGF and Other Angiogenic Factors under Hypoxia
Source: PLoS One. 2006 Dec 27;1(1):e116. doi: 10.1371/journal.pone.0000116 (PMC1762435; doi:10.1371/journal.pone.0000116)
Supplement: Table S1 — RNAhybrid, miRanda, and FindTar were used to predict potential binding sites of each human miRNA. The sites found by two or all of these three software were chosen as putative binding sites for miRNAs (all these sites were predicted by FindTar). Free energy is calculated by RNAcofold. (0.14 MB DOC) [file pone.0000116.s001.doc]

Table S1 miRNA binding sites in human VEGF 3’-UTR

| **MicroRNA** | **Location** | **Free Energy (ΔG)** | **Software** |
| --- | --- | --- | --- |
| *hsa-miR-205* | 3-18 | -23.9 | RNAhybrid |
| *hsa-miR-125a* | 5-32 | -28.6 | RNAhybrid |
| *hsa-miR-526b* | 9-32 | -30.5 | miRanda, RNAhybrid |
| *hsa-miR-378* | 11-40 | -31.3 | RNAhybrid |
| *hsa-miR-518c** | 121-143 | -22 | miRanda, RNAhybrid |
| *hsa-miR-205* | 138-157 | -21.4 | miRanda, RNAhybrid |
| *hsa-miR-93* | 166-183 | -24.1 | miRanda, RNAhybrid |
| *hsa-miR-519a* | 161-183 | -20.9 | miRanda, RNAhybrid |
| *hsa-miR-17-5p* | 165-184 | -27.5 | miRanda, RNAhybrid |
| *hsa-miR-18a* | 165-184 | -27 | miRanda, RNAhybrid |
| *hsa-miR-18b* | 167-184 | -25.1 | miRanda, RNAhybrid |
| *hsa-miR-20a* | 166-184 | -25.9 | miRanda, RNAhybrid |
| *hsa-miR-20b* | 166-184 | -29.3 | miRanda, RNAhybrid |
| *hsa-miR-106b* | 166-184 | -24.6 | miRanda, RNAhybrid |
| *hsa-miR-106a* | 162-184 | -25.9 | RNAhybrid |
| *hsa-miR-519d* | 163-184 | -21.8 | miRanda, RNAhybrid |
| *hsa-miR-520g* | 163-185 | -25 | miRanda, RNAhybrid |
| *hsa-miR-520h* | 165-185 | -23.9 | miRanda, RNAhybrid |
| *hsa-miR-373** | 161-187 | -20.7 | miRanda |
| *hsa-miR-492* | 162-190 | -21.6 | RNAhybrid |
| *hsa-miR-127* | 163-191 | -25.7 | miRanda, RNAhybrid |
| *hsa-miR-422a* | 164-191 | -23.1 | RNAhybrid |
| *hsa-miR-422b* | 164-191 | -24.7 | RNAhybrid |
| *hsa-miR-326* | 168-194 | -28.6 | miRanda, RNAhybrid |
| *hsa-miR-431* | 183-202 | -21.3 | RNAhybrid |
| *hsa-miR-122a* | 172-205 | -23.9 | RNAhybrid |
| *hsa-miR-515-3p* | 195-217 | -23.1 | miRanda, RNAhybrid |
| *hsa-miR-542-5p* | 201-221 | -20.2 | miRanda |
| *hsa-miR-425* | 205-222 | -31.3 | miRanda, RNAhybrid |
| *hsa-miR-409-5p* | 205-232 | -27.9 | RNAhybrid |
| *hsa-miR-210* | 216-239 | -23.4 | miRanda, RNAhybrid |
| *hsa-miR-517b* | 211-239 | -24.4 | RNAhybrid |
| *hsa-miR-517a* | 211-240 | -25.4 | miRanda, RNAhybrid |
| *hsa-miR-517c* | 212-240 | -22.9 | miRanda |
| *hsa-miR-127* | 238-261 | -21.8 | RNAhybrid |
| *hsa-miR-520c* | 239-267 | -20.4 | miRanda |
| *hsa-miR-107* | 253-282 | -20.2 | RNAhybrid |
| *hsa-miR-16* | 259-283 | -25.6 | miRanda, RNAhybrid |
| *hsa-miR-195* | 260-283 | -24.5 | miRanda, RNAhybrid |
| *hsa-miR-503* | 264-283 | -21.4 | RNAhybrid |
| *hsa-miR-488* | 304-323 | -25.2 | miRanda, RNAhybrid |
| *hsa-miR-520a** | 305-324 | -20.6 | miRanda |
| *hsa-miR-520d** | 304-334 | -21 | miRanda, RNAhybrid |
| *hsa-miR-199a* | 454-473 | -22.2 | RNAhybrid |
| *hsa-miR-214* | 469-489 | -20.9 | miRanda, RNAhybrid |
| *hsa-miR-199a** | 468-490 | -21.7 | miRanda, RNAhybrid |
| *hsa-miR-424* | 465-491 | -20.4 | miRanda |
| *hsa-miR-27a* | 469-492 | -21.2 | miRanda, RNAhybrid |
| *hsa-miR-510* | 478-500 | -21.3 | miRanda |
| *hsa-miR-150* | 484-506 | -25.5 | miRanda, RNAhybrid |
| *hsa-miR-339* | 520-536 | -21.9 | miRanda, RNAhybrid |
| *hsa-miR-518c** | 504-536 | -24.91 | RNAhybrid |
| *hsa-miR-10a* | 517-537 | -20.7 | RNAhybrid |
| *hsa-miR-502* | 514-537 | -24.3 | miRanda, RNAhybrid |
| *hsa-miR-502* | 603-628 | -23.8 | miRanda, RNAhybrid |
| *hsa-miR-135b* | 608-631 | -23 | miRanda, RNAhybrid |
| *hsa-miR-135a* | 607-631 | -21.2 | miRanda, RNAhybrid |
| *hsa-miR-193a* | 607-632 | -22.8 | miRanda, RNAhybrid |
| *hsa-miR-518c** | 612-633 | -23.1 | RNAhybrid |
| *hsa-miR-331* | 620-641 | -25.8 | miRanda, RNAhybrid |
| *hsa-miR-18a** | 624-644 | -23.1 | RNAhybrid |
| *hsa-miR-516-3p* | 750-778 | -21.9 | RNAhybrid |
| *hsa-miR-431* | 766-786 | -22.6 | miRanda, RNAhybrid |
| *hsa-miR-511* | 766-787 | -22.5 | RNAhybrid |
| *hsa-miR-124a* | 770-800 | -23.2 | RNAhybrid |
| *hsa-miR-330* | 768-800 | -24 | miRanda, RNAhybrid |
| *hsa-miR-189* | 823-845 | -23.2 | miRanda |
| *hsa-miR-130b* | 821-847 | -21.5 | RNAhybrid |
| *hsa-miR-515-3p* | 830-847 | -22 | RNAhybrid |
| *hsa-miR-302d* | 825-848 | -20.2 | miRanda |
| *hsa-miR-372* | 821-848 | -27.6 | RNAhybrid |
| *hsa-miR-373* | 825-848 | -25.7 | miRanda, RNAhybrid |
| *hsa-miR-519e* | 823-848 | -22.7 | RNAhybrid |
| *hsa-miR-520d* | 827-848 | -24.6 | miRanda, RNAhybrid |
| *hsa-miR-34c* | 825-850 | -25.9 | RNAhybrid |
| *hsa-miR-34a* | 831-850 | -27.2 | RNAhybrid |
| *hsa-miR-449* | 823-850 | -24.7 | miRanda, RNAhybrid |
| *hsa-miR-34b* | 826-851 | -24.5 | RNAhybrid |
| *hsa-miR-196b* | 830-851 | -21.6 | miRanda, RNAhybrid |
| *hsa-miR-196a* | 830-851 | -21.4 | miRanda |
| *hsa-let-7b* | 832-852 | -21.6 | RNAhybrid |
| *hsa-miR-30a-3p* | 832-855 | -23.2 | RNAhybrid |
| *hsa-miR-378* | 842-864 | -29 | miRanda, RNAhybrid |
| *hsa-miR-484* | 847-869 | -21.3 | miRanda, RNAhybrid |
| *hsa-miR-524* | 856-878 | -21 | miRanda, RNAhybrid |
| *hsa-miR-525** | 856-878 | -20.2 | miRanda, RNAhybrid |
| *hsa-miR-516-3p* | 889-915 | -23.1 | RNAhybrid |
| *hsa-miR-510* | 1004-1027 | -20.9 | RNAhybrid |
| *hsa-miR-140* | 1050-1069 | -20.1 | miRanda, RNAhybrid |
| *hsa-miR-485-3p* | 1080-1103 | -23.6 | RNAhybrid |
| *hsa-miR-485-3p* | 1085-1105 | -24.2 | RNAhybrid |
| *hsa-miR-483* | 1084-1112 | -20.1 | RNAhybrid |
| *hsa-miR-492* | 1115-1138 | -21.2 | RNAhybrid |
| *hsa-miR-455* | 1141-1162 | -21.9 | RNAhybrid |
| *hsa-miR-486* | 1150-1176 | -22.5 | miRanda, RNAhybrid |
| *hsa-miR-517** | 1174-1197 | -21.9 | miRanda, RNAhybrid |
| *hsa-miR-519e** | 1175-1198 | -22.8 | RNAhybrid |
| *hsa-miR-516-3p* | 1184-1199 | -22.4 | RNAhybrid |
| *hsa-miR-346* | 1164-1203 | -33.6 | RNAhybrid |
| *hsa-miR-508* | 1410-1433 | -21.9 | miRanda, RNAhybrid |
| *hsa-miR-134* | 1413-1435 | -23.6 | RNAhybrid |
| *hsa-miR-320* | 1411-1442 | -21.4 | RNAhybrid |
| *hsa-miR-491* | 1484-1507 | -22.47 | RNAhybrid |
| *hsa-miR-409-3p* | 1494-1516 | -21.6 | miRanda |
| *hsa-miR-200a** | 1502-1530 | -20.9 | RNAhybrid |
| *hsa-miR-361* | 1605-1625 | -20.4 | miRanda |
| *hsa-miR-182* | 1612-1641 | -21 | miRanda, RNAhybrid |
| *hsa-miR-485-5p* | 1779-1804 | -23.7 | miRanda, RNAhybrid |
| *hsa-miR-217* | 1801-1833 | -20.3 | RNAhybrid |

RNAhybrid, miRanda, and FindTar were used to predict potential binding sites of each human miRNA. The sites found by two or all of these three software were chosen as putative binding sites for miRNAs (all these sites were predicted by FindTar). Free energy is calculated by RNAcofold.
