# Supplementary figures and images for: MiRNA-Directed Regulation of VEGF and Other Angiogenic Factors under Hypoxia
Source: PLoS One. 2006 Dec 27;1(1):e116. doi: 10.1371/journal.pone.0000116 (PMC1762435; doi:10.1371/journal.pone.0000116)

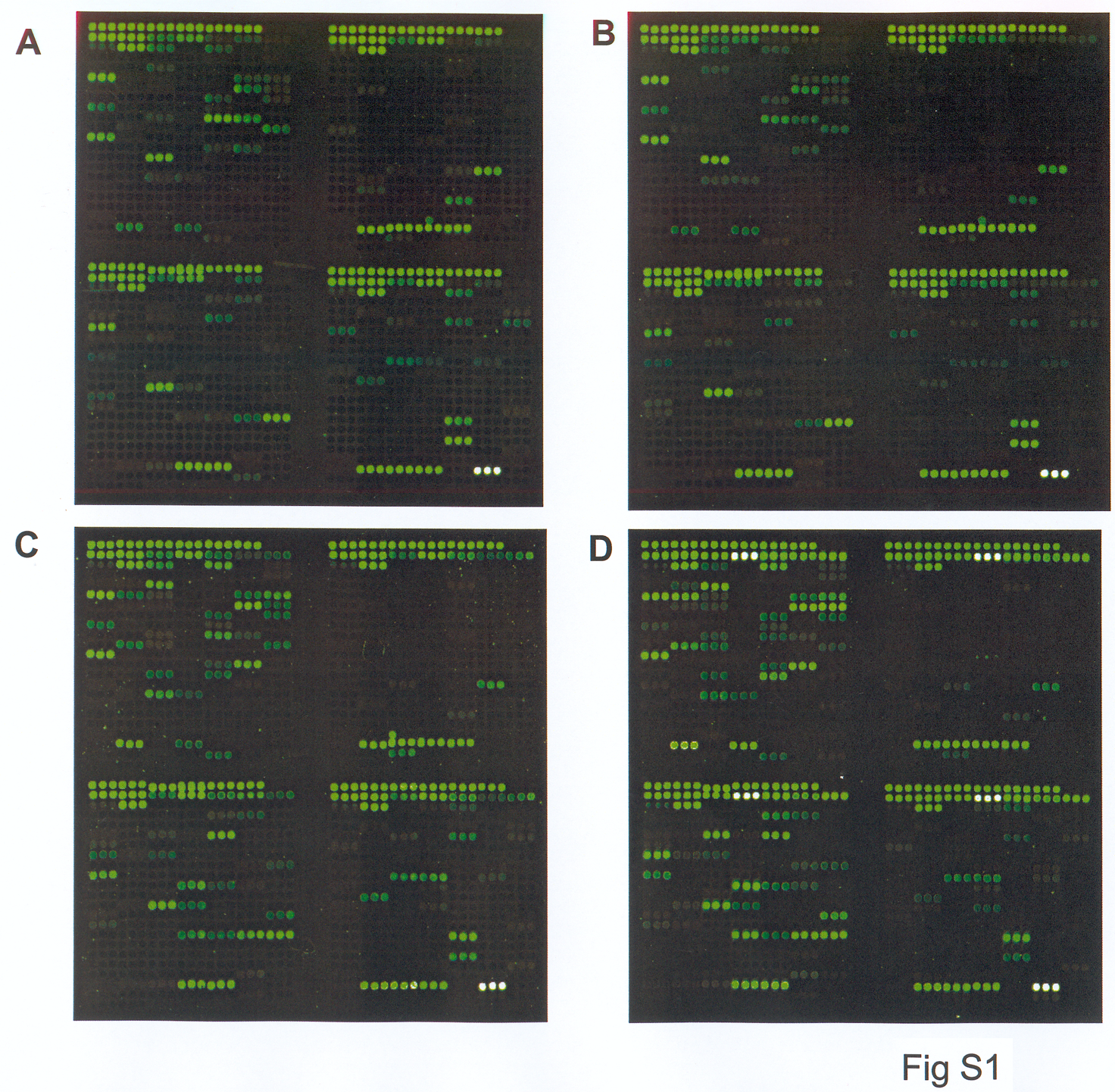

Supplement: Figure S1 — Image of miRNA microarray. miRNA array analysis was performed with mRNA samples from hypoxia-induced CNE cells (A and B) and non-induced CNE cells (C and D). An miRNA microarray chip containing 509 probes in triplicate was used in the analysis. (6.39 MB TIF) [file pone.0000116.s002.tif]
